# Protection against Severe Illness versus Immunity—Redefining Vaccine Effectiveness in the Aftermath of COVID-19

**DOI:** 10.3390/microorganisms11081963

**Published:** 2023-07-31

**Authors:** Renuka Roche, Nouha H. Odeh, Abhay U. Andar, Mohan E. Tulapurkar, Joseph A. Roche

**Affiliations:** 1 Occupational Therapy Program, School of Health Sciences, College of Health and Human Services, Eastern Michigan University, Ypsilanti, MI 48197, USA; rroche@emich.edu; 2 Ph.D. Program in Immunology and Microbiology, Department of Biochemistry, Microbiology & Immunology, School of Medicine, Wayne State University, Detroit, MI 48201, USA; gg7297@wayne.edu; 3 Baltimore County, Translational Life Science Technology, University of Maryland, Rockville, MD 20850, USA; dr.abhay.andar@gmail.com; 4 Division of Pulmonary and Critical Care Medicine, University of Maryland School of Medicine, Baltimore, MD 21201, USA; 5 Physical Therapy Program, Department of Health Care Sciences, Eugene Applebaum College of Pharmacy and Health Sciences, Wayne State University, Detroit, MI 48201, USA

**Keywords:** SARS-CoV-2, immunity, cytokines, infectious diseases, public health

## Abstract

Anti-SARS-CoV-2 vaccines have played a pivotal role in reducing the risk of developing severe illness from COVID-19, thus helping end the COVID-19 global public health emergency after more than three years. Intriguingly, as SARS-CoV-2 variants emerged, individuals who were fully vaccinated did get infected in high numbers, and viral loads in vaccinated individuals were as high as those in the unvaccinated. However, even with high viral loads, vaccinated individuals were significantly less likely to develop severe illness; this begs the question as to whether the main effect of anti-SARS-CoV-2 vaccines is to confer protection against severe illness or immunity against infection. The answer to this question is consequential, not only to the understanding of how anti-SARS-CoV-2 vaccines work, but also to public health efforts against existing and novel pathogens. In this review, we argue that immune system sensitization-desensitization rather than sterilizing immunity may explain vaccine-mediated protection against severe COVID-19 illness even when the SARS-CoV-2 viral load is high. Through the lessons learned from COVID-19, we make the case that in the disease’s aftermath, public health agencies must revisit healthcare policies, including redefining the term “vaccine effectiveness.”

## 1. Introduction

On 5 May 2023, the World Health Organization (WHO) declared that coronavirus disease 2019 (COVID-19), which is linked to infection with the severe acute respiratory syndrome coronavirus 2 (SARS-CoV-2) [1] is no longer a public health emergency of international concern (PHEIC) [2,3,4]. The WHO cautioned that the end of COVID-19′s PHEIC status was not a license to stop being vigilant against the disease, but rather that COVID-19 should now be managed by local public health agencies along with other common infectious diseases [2,3,4]. The WHO justified its decision on the basis of data, which indicate that the proportion of infections leading to severe illness and deaths from COVID-19 have satisfactorily declined since the PHEIC was first declared in January of 2020 [2,3,4]. This is indeed a cause for celebration, given how overwhelmed healthcare delivery systems were during the early waves of COVID-19 [5,6,7,8,9,10,11,12,13]. The accelerated development, testing, and deployment of anti-SARS-CoV-2 vaccines (a.k.a., COVID-19 vaccines), which were deemed safe and effective by various local regulatory bodies, and ratified by the WHO and other global public health agencies, have played a pivotal role in reducing the likelihood of severe illness and death from COVID-19 [14,15]. Based on data from 185 countries, computational models suggest that during just the first year after the deployment of COVID-19 vaccines, there was about a 63% reduction in total deaths [16]. While celebrating the end of the COVID-19 PHEIC is warranted, it is important that the WHO and other health agencies critically assess the public health management of COVID-19 in order to more rapidly and effectively contain infectious disease outbreaks that might occur in the future [17].

In this review, we present two main lessons learned from the COVID-19 pandemic. First, we present information on the pathophysiology of COVID-19 and discuss how pathogens similar to SARS-CoV-2 might be causing severe illness and death. Second, we review the evidence on COVID-19 vaccines and make the case that immune system sensitization followed by desensitization to reduce severe outcomes (without necessarily reducing infections) might be an acceptable benchmark for vaccine effectiveness compared to sterilizing immunity (achieving an absolute reduction in infections).

## 2. SARS-CoV-2—Killer Virus or Just a Trigger for Kitchen Sink Inflammation?

SARS-CoV-2, which causes COVID-19, is a non-segmented, single-stranded, positive-sense RNA virus from the β genus of coronaviruses [18,19]. SARS-CoV-2 infection was first identified in Wuhan, Hubei Province, China in December of 2019, and was initially characterized as virally-induced pneumonia by clinicians before it was finally isolated in bronchoalveolar lavage fluid from patients [19,20]. The clinical presentation of COVID-19 ranges from asymptomatic infection to mild respiratory symptoms to severe viral pneumonia [21].

**Figure 1:**
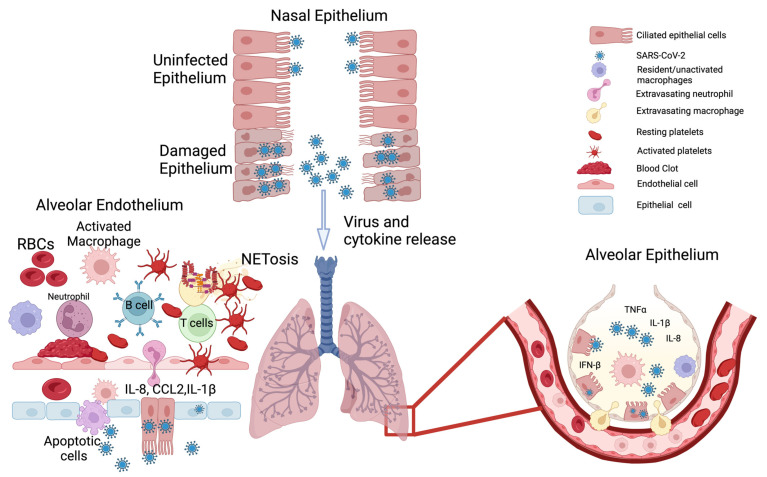
Pathophysiology of COVID-19 and “kitchen sink inflammation”.

While SARS-CoV-2 initially infects and compromises the respiratory system, it also induces multiorgan dysfunction and damage [22,23,24]. One of the main factors implicated in COVID-19 multiorgan failure is a massive release of proinflammatory cytokines—formally known as cytokine release syndrome (CRS) but better known by the colloquial term “cytokine storms” [25,26]. The inflammatory reaction in COVID-19 is due to the overactivation of multiple cellular subtypes in the human body [27]. Inhaled viral particles bind to epithelial cells in the nasal mucosa or travel down the nasopharyngeal tract to reach the more distal areas of the airway. The effect of the virus in eliciting an inflammatory response through different cell types is detailed below, and this gives us an idea of how the virus wreaks havoc on homeostasis in the host. Local and systemic inflammation followed by systemic disruption of homeostasis leads to multiorgan symptoms, multiorgan damage, and the high case fatality rate associated with SARS-CoV-2 infection (Figure 1).

### 2.1. Epithelial System

The epithelial cells are the first line of defense against invading pathogens [28]. The viral particles encounter different kinds of epithelial cells as they travel from the nose and mouth, which are the most common points of entry, all the way down to the alveolar sacs—the sites of gaseous exchange (Figure 1) [29]. The nasal mucosa has different cell types, such as ciliated epithelial cells, mucous cells, and basal cells [30]. These cells express angiotensin converting enzyme-2 (ACE2) and the transmembrane protein serine protease-2 (TMPRSS2) on their plasma membranes [31,32,33]. ACE2, TMPRSS2, and other plasma membrane proteins aid in the docking of SARS-CoV-2 onto host cells and facilitate endocytosis of the virus [32]. Once the virus has entered host cells, the virus releases its genetic material into the cytoplasm and hijacks the host’s cellular machinery to produce more viral particles [34,35]—i.e., the cell is now infected with the virus. The release of viral particles from cells has been detected as early as one hour post-infection; however, 6–8 h post-infection is when significant viral load has been detected and infection of neighboring cells and loss of ciliated epithelium has been observed [36]. The loss of cilia on the epithelium results in reduced mucociliary clearance, which leads to increased local infection and progression of the disease [37,38]. This loss of clearance has been implicated in increased COVID-19 severity in individuals with pre-existing pulmonary inflammatory conditions like asthma [39], cystic fibrosis [40], and chronic obstructive pulmonary disease (COPD), where ciliary function is already impaired [41]. In nasal epithelial cells, there is a delayed or a muted interferon response that is observed after infection. The levels of type I (interferon alpha, beta) and type III (interferon lambda) interferons are lower in severe COVID-19 compared to mild cases [42,43,44].

As the virus moves down the airway, it encounters the bronchial epithelial cells, the mucus cells, and the club cells [30] (Figure 1). In the middle and lower airway, ciliated epithelial cells that become infected lose their cilia and have a denuded appearance, and the levels of proinflammatory signals in these cells correlate with the disease severity [45].

When the virus finally reaches the alveoli (the distal-most regions of the airway) where the epithelium, endothelium, and blood cells interface and enable gas exchange [46], it activates numerous signaling cascades in multiple cell types, which cause the most damage and destruction [47] (Figure 1). In alveoli, the virus primarily infects alveolar type 2 epithelial cells (a.k.a., AT2 cells or type 2 pneumocytes), potentially due to the abundance of ACE2 on their surface [48]. Infection of type 2 pneumocytes seems to be the initial step that triggers a domino of inflammatory signals [49]. It has been shown that alveolar type 1 cells can also be infected, but to a lesser extent [50]. The infection and damage of alveolar pneumocytes results in a cascade of physiological changes, beginning with an increased production of proinflammatory cytokines, such as interleukin (IL)-1 (IL-1), IL-6, IL-8, tumor necrosis factor-alpha (TNF-α), and elevated levels of C-reactive protein (CRP) and D-dimer [51,52,53,54,55]. These inflammatory cytokines result in recruitment of immune cells—mainly neutrophils and macrophages to loci of inflammation. The recruitment of immune cells further exacerbates the situation, as it results in the loss of barrier function of the underlying endothelial layer [56,57]. Alveolar macrophages, which are recruited to sites of damage have been shown to produce various chemokines, such as CCL2, CCL3, CCL7, CCL8, CCL13, CCL20, and cytokines, such as CXCL1, CXCL3, and CXCL10 [58,59]. Among these chemokines, CCL2 and CCL3 attract more monocytes and macrophages to the alveoli and induce CXCR1 gene expression in them; this promotes the production of tissue-damaging, proinflammatory reactive oxygen species [60]. The sum effect is largescale destruction of epithelial tissue, which promotes the recruitment of neutrophils that try to resolve the situation by forming neutrophil extracellular traps (NET) [61]. The heightened cellular signaling and subsequent tissue damage described above leads to alveolar flooding with the interstitial fluid and the blood, which leads to progressive hypoxia. Thus, the replication of the virus in the alveoli results in the progression towards acute respiratory distress syndrome (ARDS), triggering an imbalance between pro-coagulation and anticoagulation (i.e., pro-fibrinolysis) pathways, plus stimulating complement activation, damage to hyaline membranes, and the formation of clots in the small and large blood vessels [62,63].

A. Nasal epithelium. The primary site of entry for SARS-CoV-2 is the nasal epithelium along with the oral mucosa. The virus binds to ciliated epithelial cells via ACE2 and is endocytosed, thus infecting the host cells. The virus uses host cell machinery to replicate and release more viral particles. The release of the viral particles results in the loss of epithelial barrier integrity, the release of inflammatory mediators, and the aggravation of symptoms. B. Alveolar epithelium. The Alveolar epithelium consists of ciliated type 2 alveolar epithelial cells (AT2) and type 1 non-ciliated (AT1) cells. Infection of the epithelial cells results in the production of a multitude of proinflammatory cytokines that promote extravasation/activation of the neutrophils and macrophages. This results in loss of barrier integrity, alveolar flooding, loss of functional surfactant, diffuse alveolar damage, and damage to the underlying endothelial cells. C. Endothelial cells. The loss of barrier integrity on the epithelial layer promotes further spread of damage to the endothelial layer. Secreted proinflammatory cytokines and chemokines recruit and activate a variety of immune cells (macrophages, neutrophils, and T and B cells). The exposed extracellular matrix also acts as a trigger for neutrophils, which undergo a process of cell death called NETosis; this leads to the aggregation and entanglement of platelets, thus promoting clot formation. Furthermore, T cells that are recruited to the site of injury can also aggravate endothelial injury. Figure created with BioRender.com.

### 2.2. Endothelial System

The endothelial cells line the innermost layers of the blood vessels (adjacent to the lumen) and tightly regulate the transport of nutrients, gases, metabolic wastes, bioactive molecules, and cells [64]. The endothelial cells lining the smaller capillaries express ACE2 and TMPRSS2, which as mentioned earlier are necessary for the host cell binding and internalization of SARS-CoV-2 [65]. The virus, on entering the endothelial cells, replicates and then disseminates through the blood stream to organs other than the lungs. The endothelial cells respond to the plethora of cytokines/chemokines that are produced by the epithelial cells upon infection. The predominant chemical signals that affect endothelial barrier function are IL-6 and TNF-α. Activation of the endothelial cells by IL-6 or TNF-α results in the production of IL-8 (a major chemoattractant for neutrophils) and monocyte chemoattractant protein (MCP-1) and in the activation of the C5a complement [66]. Indeed, plasma IL-6 levels in patients with COVID-19 correlate with the disease severity [67]. The recruitment of overly activated immune cells, a hallmark of COVID-19, can result in endothelial cell death, which promotes vascular leak and initiation of microfoci for clot formation [68,69]. Activated endothelial cells can release large amounts of von Willebrand factor (VWF) and factor VIII, which play an active role in clot formation [70]. In addition to stimulating the formation of fibrin, endothelial cells also secrete plasminogen activator inhibitor-1 (PAI-1), which inhibits clot dissolution [71]. During the initial stage of infection, the loss of contact with the surrounding cells results in the formation of circulating endothelial cells (CECs), which can travel from one tissue to another. During the course of infection, there is a loss of endothelial barrier integrity, which results in fluid accumulation in the alveolar and pleural spaces. This is a result of the upregulation of interleukin 2 receptor (IL-2R) on the endothelial cells and the increased release of IL-2 from the activated T cells [72]. Thus, direct viral infection or indirect activation of endothelial cells causes a loss of barrier function, which then results in fluid accumulation, increased extravasation of immune cells, widespread microthrombosis, diffuse fibrin deposits, and secretion of PAI-1 that prevents clot dissolution—all working together to increase the likelihood of thromboembolisms [73,74] (Figure 1).

### 2.3. The Mismatch between Viral Load and Symptom Severity

In Section 2, we attempted to answer the question as to whether SARS-CoV-2 is a killer virus or just a trigger for “kitchen sink inflammation”. The related expressions “to throw everything but the kitchen sink” or “to throw the kitchen sink” imply doing everything possible to address a problem, regardless of whether the solutions are likely to be good or bad [75,76]. Due to the novelty of SARS-CoV-2 to the human immune system, when people were first infected, the immune response was indeed comparable to throwing an inflammatory kitchen sink at the viral invader, resulting in cell-signaling storms [25,26,47,55,77,78,79,80,81,82,83]. The fact that some individuals with high viral loads presented with minimal symptoms, and some individuals with severe symptoms had low viral loads suggest that, SARS-CoV-2 by itself is not a killer virus, but rather, it is a nonspecific and overly aggressive inflammatory response, which causes the most harm [84,85,86,87,88]. This is not to diminish the fact that SARS-CoV-2 infection has been implicated in the deaths of nearly seven million people, chronic illness in 10–20% of the survivors, and about 800 million cumulative cases in just over three years [89,90]. However, the available data point towards severe illness and deaths being linked to an inflammatory response, which overwhelms the host, rather than widespread virus-mediated killing of cells in the respiratory system and other vital systems of the body [88]. Interestingly, an overly aggressive immune response, rather than the pathogen itself, might underlie severe illness in various infectious diseases [91]. Therefore, public health agencies must invest resources in finding safe, effective, practical, and standardized therapies, which can work to combat a wide range of existing and novel pathogens. In hindsight, one of the simplest public health strategies to blunt the impact of SARS-CoV-2 would have been to assume that it would behave just like its predecessor SARS-CoV (implicated in the SARS outbreak of the early 2000s) and amplify the importance of commonsense preventative healthcare measures (e.g., hand hygiene, mask wearing, and avoiding unnecessary social activities) until safe and effective pharmacological measures were available [92,93,94].

## 3. Calming the Inflammatory Storm by Conditioning the Immune System

For over a century, vaccines have served as a beacon of hope against infectious diseases [95,96]. From mitigating the severity of various infectious diseases to completely eradicating certain diseases in populations, vaccines are easily one of mankind’s greatest innovations in the realm of public health [97,98]. Vaccines come in a variety of forms such as live attenuated, inactivated, recombinant, toxoid, viral vector, and the recently developed messenger RNA (mRNA vaccines) [95,99]. These formulations all follow the same basic principle of exploiting the human immune system by exposing it to an innocuous form, portion, or product of a pathogen to induce long-lasting protection against the pathogen [100]. The benefits of this practice are reaped at an individual and population level [97,98]. The individual, upon exposure to the real pathogen post-vaccination, avoids severe disease outcomes due to his or her immune system being primed against it. On a population level there exists the goal of herd immunity, where entire communities are protected against a pathogen due to the high percentage of individuals who are vaccinated and/or have been exposed to natural infection [101,102]. If the fraction of the population that is vaccinated is low, then those who are not eligible for vaccination due to age, those who are immunocompromised, and those who might be particularly vulnerable to disease complications may not benefit from community-level protection. It is imperative that a proper understanding of vaccines is reached to maximize their effectiveness and attain herd immunity whenever possible for the maximum benefit to individuals and societies. This is especially relevant considering the recent COVID-19 pandemic, where vaccination has played a critical role in disease mitigation.

### 3.1. How the Most Common COVID-19 Vaccines Work

As highlighted earlier, severe cases of COVID-19 can trigger sepsis, hypoxemia, pneumonia, and tissue damage [21,103]. In line with what is expected of respiratory viruses, SARS-CoV-2 is transmitted mainly via the respiratory route by droplets and aerosolized viral particles and also possesses an incubation period of approximately 5–12 days, making the control of its spread challenging [18,19,20].

**Figure 2:**
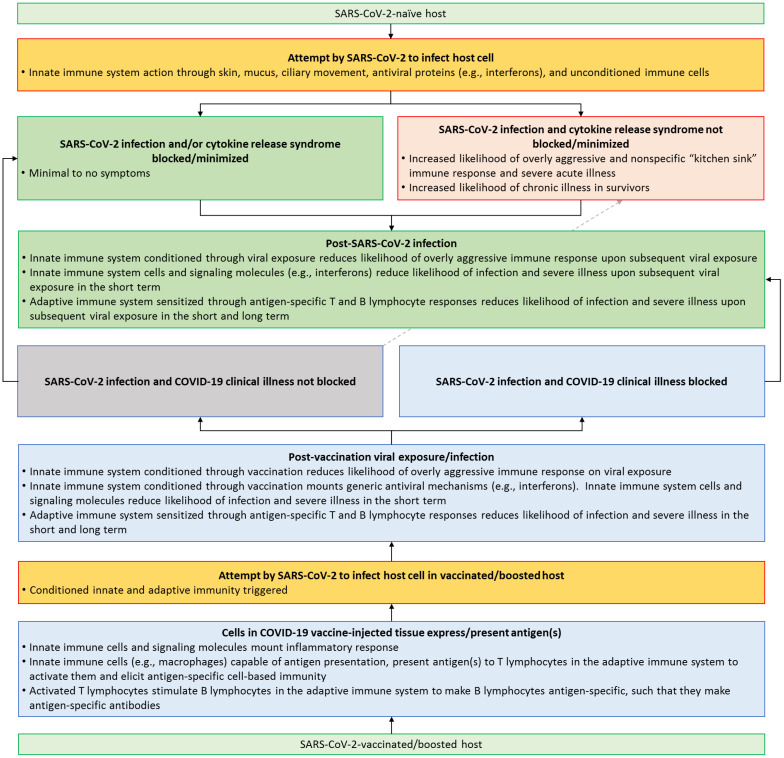
The role of vaccine-induced immune system conditioning in reducing COVID-19 illness severity.

COVID-19 vaccines were in development within less than six months since the beginning of the outbreak, with public health agencies and industry partners around the world committing vast resources to vaccine development, testing, and deployment [104]. Out of these efforts emerged the novel mRNA vaccines, such as those produced by Moderna (Spikevax) and Pfizer-BioNTech (Comirnaty) [99,104]. These mRNA vaccines consist of a segment of mRNA, which has the genetic code to make the SARS-CoV-2′s spike protein. Since naked mRNA is not stable, it was packaged in a lipid (a basic chemical component of fats and oils) coat to stabilize the structure and allow it to be readily delivered into cells [99,105,106,107]. Based on the established understanding of conserved cellular biological processes, we can assume that, once the mRNA-containing lipid spheres are inside a host cell, such as a muscle fiber in the deltoid muscle (the muscle that is most commonly injected), the mRNA information is used by the host cell ribosomes to assemble the amino acid sequence for the spike protein through a process called protein translation [106,107]. After translation, spike protein molecules are processed and presented as an antigen at the surface of the host cell, thereby activating both innate and adaptive immune responses, which confer immune memory [105,106,107] (Figure 2). Another anti-SARS-CoV-2 vaccine strategy that was widely employed, involved the use of DNA packaged in a harmless adenovirus [107,108]. DNA within the adenovirus contained the code needed for inoculated host cells to generate mRNA in their nuclei through DNA transcription; the mRNA would then exit the nuclei and bind to the ribosomes, and from that point on, the assembling and presentation of spike protein molecules was similar to that of mRNA vaccines [107].

The recognition of “self” from “non-self” is a fundamental aspect of immunity, and this function serves an important role in protecting the body from disease-causing elements [109]. Vaccination takes advantage of this by introducing a harmless version of the disease-causing pathogen, allowing the body to recognize and remember it. There are two phases of immunity that are activated upon infection or inoculation through vaccination: Innate immunity and adaptive immunity [110,111,112] (Figure 2). Innate immunity is the first line of defense and is activated when pattern recognition receptors detect foreign material or damage, which are known as pathogen- associated molecular patterns (PAMPs) and damage-associated molecular patterns (DAMPs) [110,111,113]. The result is the release of proinflammatory molecules, such as cytokines, which recruit more immune cells to the site of vaccination [110,111,113] (Figure 2). Shortly after this initial response, the adaptive immune system is activated (Figure 2). The adaptive immune system is critical for the control of viral infections, especially in the long term upon re-exposure to the same virus (Figure 2). The main players in the adaptive immune response are T and B lymphocytes, which possess a range of functions that include killing infected cells, activating other lymphocytes, producing antibodies, and generating memory cells [112] (Figure 2). With the production of memory cells and antibodies against SARS-CoV-2 through vaccination, the expected outcome in a vaccinated individual who was subsequently exposed to the actual virus, was a decreased likelihood of infection, decreased viral load if infected, reduced ability to transmit infection, and protection against severe illness (Figure 2). These predictions were indeed borne out by initial clinical trials, thus prompting emergency use authorization and approval of certain mRNA and DNA-adenovirus vaccines [108,114,115].

### 3.2. COVID-19 Vaccination—Sterilizing Immunity or Protection against Severe Illness?

As discussed earlier, vaccination against COVID-19 was predicted to confer sterilizing immunity—i.e., reduce the likelihood of infection and therefore transmission [116]. However, as real-world data from community vaccination programs emerged, it became clear that even those considered to be fully vaccinated (those who completed the required vaccination schedule for their age and health status) were still being infected in high numbers. Infections in individuals who were considered fully vaccinated were called “breakthrough cases” [117,118,119]. The incidence of breakthrough cases increased as new variants and subvariants of SARS-CoV-2 emerged and created waves of infections that swept across continents [120,121]. The first and earliest variant was identified in the UK and called Alpha, the second was identified in South Africa and called Beta, and the third was found in Brazil and called Gamma; Eta and Delta were the fourth and fifth variants, which were identified in the UK and India, respectively [120]. The Omicron variant was identified in Botswana and is the most highly mutated strain, with twice as many mutations than the Delta variant [121,122]. The incidence of breakthrough cases during the Delta variant wave was high; however, despite the Delta variant being characterized as more aggressive in its symptomatology than prior strains [123,124], the disease outcome in vaccinated individuals was better than in those who were unvaccinated [125,126,127]. Intriguingly, during the Delta variant wave of COVID-19, viral loads in fully vaccinated individuals were as high as those seen in unvaccinated individuals, indicating that vaccination was reducing the risk of severe illness without reducing host cell viral infection and without blunting the production of viral particles in infected individuals [117,118,128,129,130,131]. During the Delta wave, it was also humbling to learn that vaccinated individuals with breakthrough infections were just as likely as unvaccinated individuals to transmit the virus to others—disturbing news at a time when public health agencies in certain countries were relaxing nonpharmacological COVID-19 mitigation measures against a backdrop of vaccine inequity based on age, geography, economics, and other factors [117,132]. The conferring of protection against severe illness and death through vaccination without blocking infection has continued through subsequent waves of COVID-19 infections caused by less aggressive variants and subvariants (e.g., Omicron) [133]. These observations are likely linked to our earlier point about how a nonspecific and overly aggressive inflammatory response to SARS-CoV-2, rather than the extent of viral infection, might be the factor underlying disease severity [88]. Based on the lessons learned from COVID-19 vaccination programs, it is imperative that public health agencies reassess the benchmarks for vaccine effectiveness, since the benefits of vaccination have medical, sociological, and economic implications that extend far beyond the mere conferring of immunity as we know it [97,134]. While aiming to obtain sterilizing, lifelong immunity through vaccination is a fair goal, settling for vaccine-mediated protection against severe illness for fast-spreading diseases like COVID-19 would be an acceptable strategy, so that, the highest number of individuals around the world are rapidly protected and negative economic impact is reduced [102,135].

The flow chart summarizes how the innate and adaptive immune systems likely play vital roles in reducing the risk of severe acute COVID-19 illness through vaccination, natural infection, and natural infection following vaccination [28,136,137,138,139]. We make the case that being up to date on COVID-19 vaccination through updated SARS-CoV-2 vaccines/boosters acts as a type of “continuing education” for the immune system. This continuing education enables the immune system to remain conditioned, such that it mounts a calm and focused response to viral exposure and does not overwhelm the host through overly aggressive inflammation. We also indicate that in rare cases even vaccinated individuals might develop severe acute illness and chronic complications (the dotted grey arrow connecting the vaccination story in the flow chart (bottom) to the natural infection story in the flow chart (top)) [140,141].

### 3.3. Could Immune System Sensitization Followed by Desensitization Explain Vaccine-Induced Protection against Severe COVID-19?

Early in the COVID-19 pandemic, it was suggested that zoonotic transmission (animal to human) from bats was the most likely source of SARS-CoV-2 and caused the first set of infections in humans [142]. Viral genomes sharing > 90% similarity to human SARS-CoV-2 were found in bats and pangolins [143,144,145,146]. As the pandemic progressed, there were reports of other animals being infected with SARS-CoV-2, raising concerns that animals could act as viral reservoirs, leading to more human infections [142]. Similar to observations in certain vaccinated and unvaccinated humans, animals that test positive for SARS-CoV-2 may have mild to no symptoms but can still serve as reservoirs/vectors of the virus [147]. These observations also support the idea that severe illness does not usually ensue if the host does not mount a nonspecific and overly aggressive inflammatory response (Figure 1 and Figure 2). Thus, it is likely that vaccination initially acts as a sensitizer to the immune system, which is naïve to SARS-CoV-2, but that through this sensitization, when an actual infection happens later, the result is a more tolerant, subdued, and conditioned inflammatory response that does not overwhelm the host [148,149,150] (Figure 2). Immune system desensitization is the process of gradually exposing an individual to an antigen so that their immune system becomes nonreactive [151]. Desensitization has been used for decades to improve clinical outcomes in people with allergies and asthma [151,152,153]. It is tempting to speculate if small doses of SARS-CoV-2 antigens that are updated to include new variants, might not only be able to act as a form of continuing education to the adaptive immune system, but also act as a way to maintain a conditioned and tolerant innate immune system, which does not overwhelm the host through inflammation when exposed to the actual pathogen [154] (Figure 2). Intriguingly, vaccine desensitization protocols have been successfully used to reduce the likelihood of allergic reactions in individuals who display signs of COVID-19 vaccine hypersensitivity [155,156,157].

### 3.4. Cutaneous Delivery of SARS-CoV-2 Vaccines as a Potential Strategy to Rapidly Vaccinate/Boost a Large Number of Individuals

In the preceding section, we presented evidence to suggest that COVID-19 vaccines offer protection to the host against severe illness and death even if they do not reduce the risk of infection. We then made the case that, if protection against severe illness rather than sterilizing immunity is an acceptable benchmark for vaccine effectiveness, it may be possible to achieve this by periodically exposing the immune system to small amounts of updated antigenic material. The logistics of administering updated vaccines to the entire global population on a semiannual or even annual basis is daunting [158]. It is in this context that cutaneous vaccine delivery systems are appealing.

The skin is a viable organ with a large concentration of antigen-presenting cells including the epidermal Langerhans cells and dermal dendritic cells [159]. The minimally invasive microneedle (MN) of cutaneous drug delivery systems reduces safety concerns associated with parenteral administration and increases patient compliance, thus making MN-enhanced transdermal delivery an attractive route for vaccine administration. Different types of antigens including inactivated whole virus, live attenuated virus, virus-like particles, recombinant bacteria, protein subunits, and plasmid DNA have been investigated to demonstrate immune responses following MN delivery. Many promising examples of MN-based vaccine delivery have appeared in recent publications [160,161], a few of which have been highlighted in this section. Antigens can be introduced into the skin using different MN approaches, including intradermal injection (similar to the Mantoux method), stratum corneum disruption by solid MN abrasion, antigen-coated MN, and dissolvable MN insertion [162].

Intradermal delivery using MNs can provide superior antigenicity, as demonstrated through studies with influenza vaccines. Using three different types of influenza vaccines (a whole inactivated influenza virus, a trivalent split-virion human vaccine, and a plasmid DNA encoding the influenza virus hemagglutinin), the MN-based system provided up to a 100-fold dose sparing in rats compared to intramuscular injection for the same induced immune response [163]. Recent clinical trials in adults showed that an MN-based delivery of influenza vaccine can induce immune responses with less antigen (9 µg) at rates comparable to those with intramuscular (15 µg) vaccination [164]. A stronger humoral immune response in the elderly was also induced with MN-based intradermal delivery compared to intramuscular injection [165]. A hollow 1.5 mm–long MN Becton Dickinson Soluvia prefilled microinjection system has recently been approved to deliver Fluzone intradermal vaccine (single 0.1 mL dose, Sanofi Pasteur) for influenza prophylaxis [94]. Other MN-based vaccines currently under investigation include a recombinant Bacillus anthracis vaccine formulated with aluminum and administered by hollow MNs [166], a live attenuated chimeric flavivirus vaccine delivered by skin microabrasion for the treatment of Japanese Encephalitis and yellow fever [167], and a smallpox DNA vaccine delivered by MN coating and skin electroporation [168]. It is therefore not surprising that MN-based delivery of COVID-19 vaccines is a topic of interest, which could potentially lead to a paradigm shift in how large numbers of people could be safely and rapidly vaccinated with fewer logistical hurdles and reduced amounts of biowaste [169].

## 4. Conclusions

We started this review by highlighting the end of the COVID-19 global health emergency, which is undoubtedly a cause for celebration. We then went on to present two main lessons learned from the COVID-19 pandemic, and how these lessons prompt all public health stakeholders (i.e., the entire global human community) to examine our assumptions about how COVID-19 makes people sick and how COVID-19 vaccines work. Paying heed to these lessons could help improve public health and better prepare global communities for future health emergencies that may arise due to infectious diseases.

## Data Availability

No new data were created or analyzed for this review paper. Therefore, data sharing is not applicable to this article.
